# A Review of Auxin Response Factors (ARFs) in Plants

**DOI:** 10.3389/fpls.2016.00047

**Published:** 2016-02-03

**Authors:** Si-Bei Li, Zong-Zhou Xie, Chun-Gen Hu, Jin-Zhi Zhang

**Affiliations:** Key Laboratory of Horticultural Plant Biology, Ministry of Education, College of Horticulture and Forestry Science, Huazhong Agricultural UniversityWuhan, China

**Keywords:** auxin, ARF, auxin response DNA elements, *Arabidopsis*, DBD domain, a type I/II Phox and Bem1p (PB1)

## Abstract

Auxin is a key regulator of virtually every aspect of plant growth and development from embryogenesis to senescence. Previous studies have indicated that auxin regulates these processes by controlling gene expression via a family of functionally distinct DNA-binding *auxin response factors* (*ARFs*). *ARFs* are likely components that confer specificity to auxin response through selection of target genes as transcription factors. They bind to auxin response DNA elements (AuxRE) in the promoters of auxin-regulated genes and either activate or repress transcription of these genes depending on a specific domain in the middle of the protein. Genetic studies have implicated various *ARFs* in distinct developmental processes through loss-of-function mutant analysis. Recent advances have provided information on the regulation of *ARF* gene expression, the role of *ARFs* in growth and developmental processes, protein–protein interactions of ARFs and target genes regulated by *ARFs* in plants. In particular, protein interaction and structural studies of ARF proteins have yielded novel insights into the molecular basis of auxin-regulated transcription. These results provide the foundation for predicting the contributions of *ARF* genes to the biology of other plants.

## Introduction

Auxins play a critical role in most major growth responses both throughout the different developmental stages of plants such as **organogenesis**, vascular tissue differentiation, apical dominance and root initiation, and tropism and on a cellular level cell in processes including extension, division, and differentiation (Guilfoyle and Hagen, 2007; Mockaitis and Estelle, 2008; Su et al., 2014). Three decades of studies have explored the rapid effects of auxin on gene expression and regulation (Di et al., 2015b). A large number of candidate genes that are potentially regulated by auxins and that may function in growth and developmental processes have been identified in *Arabidopsis* and other plant species (Rosado et al., 2012; Liu et al., 2014b; Di et al., 2015b; Guilfoyle, 2015). Among these genes, members of the *auxin response factors* (*ARF*) family have been suggested to play a key role in regulating the expression of auxin response genes (Liscum and Reed, 2002). To date, 22 *ARF* genes and one pseudogene have been isolated from *Arabidopsis* (Liscum and Reed, 2002; Guilfoyle and Hagen, 2007). *ARF* genes are expressed in dynamic and differential patterning during development, and genetic studies have shown that individual *ARFs* control distinct developmental processes (Rademacher et al., 2012). Members of the ARF family of proteins contain domains associated with DNA binding, transcriptional activation or repression, and protein-protein interactions during auxin perception and signaling processes (Guilfoyle and Hagen, 2007; Di et al., 2015a). Recently, the *ARF* gene family has been also investigated in several plants based on the recent release of the genome such as citrus, *Medicago truncatula* and *Gossypium raimondii* using both bioinformatics and molecular analyses (Li et al., 2015; Shen et al., 2015a; Sun et al., 2015). More importantly, a considerable amount of new information has been obtained regarding the mechanisms that control ARF protein activities, and gene expression profiles. This review will focus on recent advances that have provided insight into the roles played by *ARFs* in regulating a variety of plant growth and development processes and the mechanisms involved in this regulation in *Arabidopsis* and other plant species.

KEY CONCEPT 1 **Organogenesis**

An adult plant consists of many specialized cell organizations: tissues and organs. Tissues consist of cells of uniform shape and specialized function, such as meristem, cortex, and phloem. Several tissues are organized together to form an organ, such as leaves, roots, flowers, and fruit. The process of initiation and development of an organ is called organogenesis.

## Molecular structure of ARF family proteins

The plants response to auxin involves changes in gene regulation (Liscum and Reed, 2002). Genes that are up-regulated or down-regulated by auxin contain AuxRE in their promoters, which bind transcription factors of the ARF family (Guilfoyle and Hagen, 2007; Mockaitis and Estelle, 2008). The identification of the AuxRE sequence led to the isolation of *Arabidopsis ARF1* (Ulmasov et al., 1997), and subsequent genetic, genomic, and molecular studies have identified 22 *ARF* genes in *Arabidopsis* (Liscum and Reed, 2002). The *ARF* gene family is a modular transcription factor family consisting of several domains that have remained conserved despite hundreds of millions of years of evolution (Finet et al., 2012). Most ARF proteins consist of an N-terminal **B3-type DNA binding domain (DBD)**, a variable middle region that functions as an activation domain (AD) or repression domain (RD), and a carboxy-terminal dimerization domain (CTD: domain III/IV), which is involved in protein–protein interactions by dimerizing with auxin/indole-3-acetic acid (Aux/IAA) family genes as well as between ARFs (Kim et al., 1997; Guilfoyle and Hagen, 2007; Piya et al., 2014). The DBD is classified as a plant-specific B3-type protein domain, but requires additional amino-terminal and carboxyterminal amino acids for efficient *in vitro* binding to TGTCTC/GAGACA site (Tiwari et al., 2003). The first four bases of the recognition site are absolutely required for ARF binding, while more variation is tolerated in the last two bases (Boer et al., 2014). The AD and RD are located just carboxyterminal to the DBD and contain biased amino acid sequences (Ulmasov et al., 1999). The AD is enriched in glutamine along with leucine (L) and serine (S) residues, while the RD is enriched in glycine (Q), leucine (L), serine (S), and proline (P) residues (Ulmasov et al., 1999). The amino acid composition of the middle region is critical in determining ARF function, with S-rich ARFs acting as transcriptional repressors and Q-rich ARFs acting transcriptional activators by protoplast transfection assays (Tiwari et al., 2003; Guilfoyle and Hagen, 2007). Five ARF proteins (ARF5/ARF6/ARF7/ARF8/ARF19) were characterized as transcriptional activators based on transient assays in transfected protoplasts, the other ARFs were classified as repressors (Ulmasov et al., 1999; Tiwari et al., 2003). A recent crystallographic study revealed that two additional domains associate with the DBDs of some ARFs, and these are a dimerization domain (DD) and a Tudor-like ancillary domain within the C-terminal region of the flanking domain (FD). The DD facilitates cooperative binding of the B3 DBD to selected AuxREs. However, the function of Tudor-like ancillary domain has not been determined (Guilfoyle and Hagen, 2012; Boer et al., 2014; Guilfoyle, 2015; Korasick et al., 2015). Not all ARFs contain the five domains described above. In addition, the III–IV region of some ARF protein may form a type I/II Phox and Bem1p (PB1) protein–protein interaction domain, which provides both positive and negative electrostatic interfaces for directional protein interaction (Guilfoyle and Hagen, 2012; Guilfoyle, 2015; Korasick et al., 2015).

KEY CONCEPT 2 **B3-type DNA binding domain (DBD)**

It is a highly conserved domain found exclusively in transcription factors (TFs) of higher plants. It consists of 100–120 residues, includes seven beta strands and two alpha helices. There are three main families of TFs that contain B3 domain in Arabidopsis: ARF Auxin response factors (ARF), Abscisic acid insensitive 3 (ABI3), and Related to ABI3/VP1 (RAV).

Since, cloning of the first *ARF* gene from *Arabidopsis* (Ulmasov et al., 1997), *ARF* genes from 15 plant species have been identified based on genome-wide analysis studies (Table 1). For example, 22 genes from tomato (Zouine et al., 2014), 25 genes from rice (Wang et al., 2007), 19 genes from sweet orange (Li et al., 2015), 24 genes from *Medicago truncatula* (Shen et al., 2015a), 47 genes from banana (Hu et al., 2015), and 39 genes from *Populus trichocarpa* (Kalluri et al., 2007) were identified. Most ARF proteins from these plant species are nuclear proteins with described protein domains consistent with previous reports on the homologous genes from *Arabidopsis* (Table 1). However, some differences in the ARF protein family were also found between *Arabidopsis* and other plant species. For example, ARF3, ARF13, and ARF17 lack Domains III/IV, and ARF23 consists of a truncated DBD only in *Arabidopsis* (Guilfoyle and Hagen, 2007). Only one pseudogene (a truncated DBD) was found in citrus plants among these plant species (Li et al., 2015), whereas a large number of truncated proteins (lacking Domains III/IV) have been found in rice (Wang et al., 2007), maize (Liu et al., 2011), banana (Hu et al., 2015), and *M. truncatula* (Shen et al., 2015a) compared with *Arabidopsis*. Interestingly, some plant species contain more *ARF* genes than *Arabidopsis* (Table 1). One explanation for the higher number of *ARF* genes encoded in these genome could be that large-scale duplication event occurred early in the evolution of these plants (Blanc et al., 2003). It is noteworthy that most information about *ARFs* function, expression, and regulation comes from studies in annual herbaceous plants such as *Arabidopsis*, rice, and tomato (Guilfoyle and Hagen, 2007; Wang et al., 2007; Kumar et al., 2011), while relatively few reports focus on other plant species.

**Table 1:** **Summary of ***ARF*** genes in 16 plant species based on genome-wide analysis**.

**Species**	**Gene No**	**Pseudogene No**	**Truncated protein No**.	**Complete protein No**.	**References**
*Arabidopsis thaliana*	23	1	3 (DBD, MR)	19 (DBD, MR, CTD)	Hagen and Guilfoyle, 2002
*Oryza sativa*	25	0	6 (DBD, MR)	19 (DBD, MR, CTD)	Wang et al., 2007
*Citrus sinensis*	19	1	3 (DBD, MR)	15(DBD, MR, CTD)	Li et al., 2015
*Solanum lycopersicum*	21	0	7 (DBD, MR)	14 (DBD, MR, CTD)	Wu et al., 2011
*Glycine max*	51	0	8 (DBD, MR)	43 (DBD, MR, CTD)	Van Ha et al., 2013
*Zea mays*	36	0	11 (DBD, MR)	25 (DBD, MR, CTD)	Liu et al., 2011
*Populus trichocarpa*	39	0	0	35 (DBD, MR, CTD)	Kalluri et al., 2007
Banana	47	0	12 (DBD, MR)	35 (DBD, MR, CTD)	Hu et al., 2015
*Brassica rapa*	31	0	4 (DBD, MR)	27 (DBD, MR, CTD)	Mun et al., 2012
*Vitis vinifera*	19	0	2 (DBD, MR)	17 (DBD, MR, CTD)	Wan et al., 2014
*Medicago truncatula*	24	0	14 (DBD, MR)	10 (DBD, MR, CTD)	Shen et al., 2015a
*Gossypium raimondii*	35	0	7 (DBD, MR)	28 (DBD, MR, CTD)	Sun et al., 2015
*Cucumis sativus*	15	0	1 (DBD, MR)	14 (DBD, MR, CTD)	Liu and Hu, 2013
*Eucalyptus grandis*	17	0	3 (DBD, MR)	14 (DBD, MR, CTD)	Yu et al., 2014
*Malus domestica*	31	0	8 (DBD, MR)	23 (DBD, MR, CTD)	Luo et al., 2014
*Carica papaya L*.	11	0	3(DBD, MR)	7 (DBD, MR, CTD)	Liu et al., 2015

## Activation, interaction, and regulatory mechanisms of *ARFs* in plants

**Figure 1:**
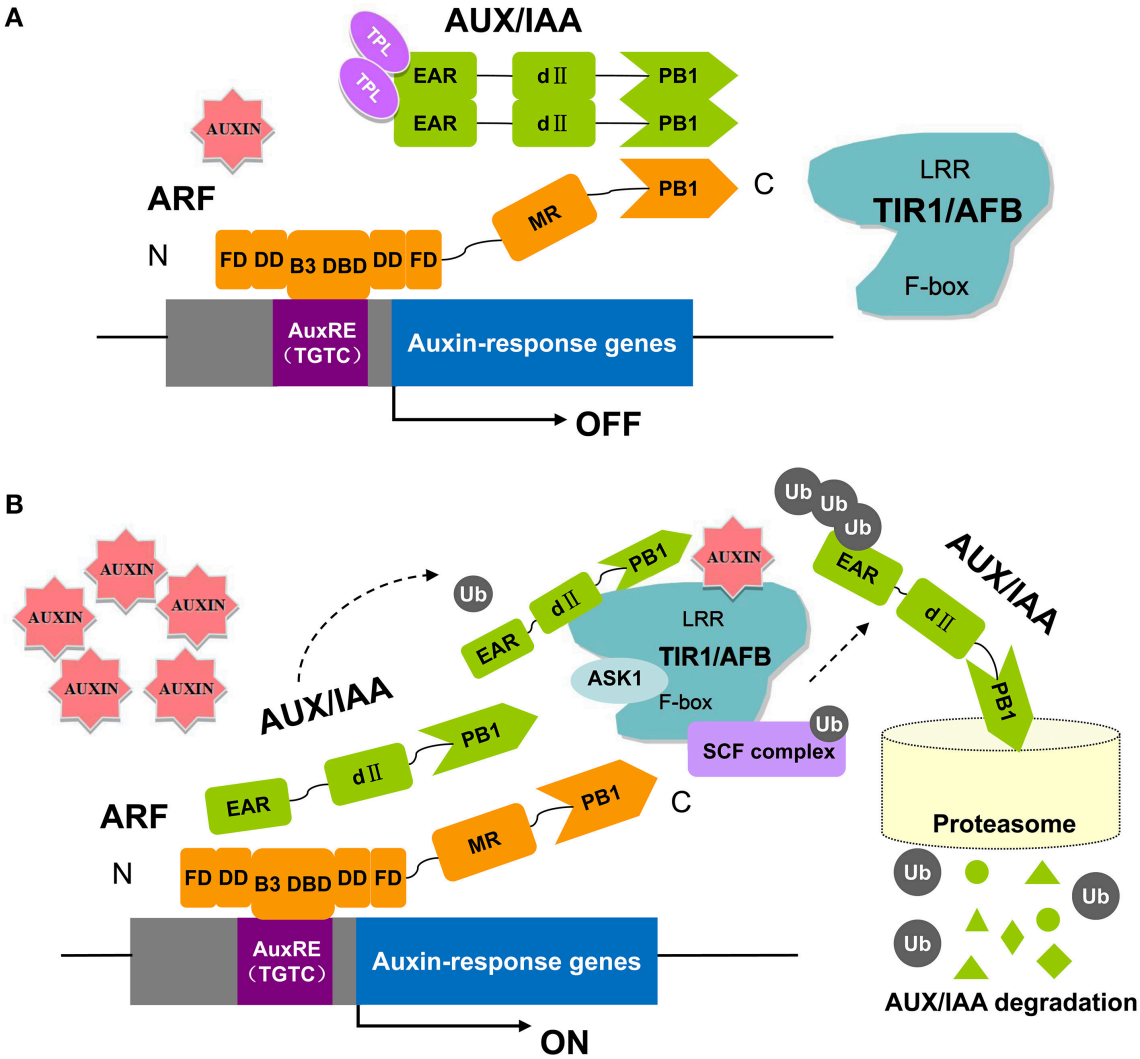
**The key components in auxin perception and signaling in ***Arabidopsis*****. ARF proteins contain a non-conserved AD or RD flanked by an N-terminal DBD (composed of a B3 domain, a dimerization domain: DD, and a Tudor-like ancillary domain within the C-terminal region of the flanking domain: FD) and a C-terminal PB1 domain (previously referred to as domain III/IV). Parts of the DD and FD are found both N-terminal and C-terminal to the B3 domain. In this pathway, the TRANSPORT INHIBITOR RESPONSE 1/AUXIN SIGNALING F-BOX proteins (TIR1/AFBs) are F-box proteins that, together with other proteins (ASK1, CUL1, RBX), form the ubiquitin protein ligase complex, SCF^TIR1^. At low auxin levels **(A)**, the Aux/IAA proteins form multimers with ARFs and recruit TPL to the chromatin. High levels of auxin **(B)** promote ubiquitination and degradation of Aux/IAAs through SCF^TIR1∕AFB^ and the 26S proteasome (Kim et al., 1997; Guilfoyle and Hagen, 2007, 2012; Szemenyei et al., 2008; Boer et al., 2014; Guilfoyle, 2015; Korasick et al., 2015; Salehin et al., 2015).

The *ARF* genes encode proteins with full-length DBDs that may recognize and compete for target sites in promoters of auxin response genes (Tiwari et al., 2003). Therefore, there has been increased interest to determining when and where these genes are expressed and what regulates their expression (Hagen and Guilfoyle, 2002; Guilfoyle and Hagen, 2007). It has long been recognized that ARFs directly bind to AuxREs in the promoters of auxin responsive genes through their DNA-binding domain (Wang and Estelle, 2014). ARF binding to AuxREs in particular requires C-terminal amino acids (Guilfoyle and Hagen, 2012). It has been proposed that the C-terminal domain enhances DNA binding by enabling ARF dimerization. Both ARF and Aux/IAA proteins contain conserved sequences near the C-terminus termed domains III and IV (Guilfoyle and Hagen, 2012). These domains mediate ARF-ARF, ARF-Aux/IAA, and Aux/IAA-Aux/IAA interactions as determined by yeast two-hybrid and **bimolecular fluorescence complementation assays** (Korasick et al., 2015). ARF regulation is well-studied, and a working model for ARF activation is now well-established (Figure 1) (Salehin et al., 2015). At low auxin levels, Aux/IAA proteins form dimers with ARFs to inhibit ARF activity by recruiting the co-repressor TOPLESS (TPL), which results in the repression of auxin-responsive genes (Figure 1A) (Szemenyei et al., 2008). At higher auxin levels, Aux/IAAs bind to the SCF^TIR1∕AFB^ complex and subsequently become ubiquitinated and degraded by the 26S proteasome. The ARF is then released and can regulate the transcription of its target auxin response genes (Figure 1B) (Wang and Estelle, 2014). Recent structural studies of ARFs have led to exciting new insight into the molecular function of the ARF-Aux/IAA pathway. Crystal structures showed that the C-terminal domains of ARF5 and ARF7 conform to a well-known PB1 domain that confers protein-protein interactions with other PB1 domain proteins through electrostatic contacts (Boer et al., 2014; Guilfoyle, 2015). Further experiments confirmed the importance of these charged amino acids in conferring ARF and Aux/IAA interactions as proposed by the crystal structure of the PB1 domain (Korasick et al., 2015). In addition to the PB1 domain, a second protein-protein interaction module that functions in ARF-ARF dimerization and facilitates DNA binding has recently been revealed from structure-function analysis and saturating binding site selection on the ARF1 and ARF5 DNA binding domains (Boer et al., 2014). These studies provide an atomic-level explanation for DNA-binding specificity in the auxin pathway.

KEY CONCEPT 3 **Bimolecular fluorescence complementation assays**

It is a technology typically used to validate protein interactions. It is based on the association of fluorescent protein fragments that are attached to components of the same macromolecular complex. Through the Visualization and analysis of the intensity and distribution of fluorescence in live cells, one can identify both the location and interaction partners of proteins of interest.

In addition to the interaction between themselves, the ARFs have also been reported to regulate and be regulated by other transcription factors (Wang and Estelle, 2014). A recent study showed that a *MYB* transcription factor (*MYB77*) interacts with the ARF7 protein and that this interaction results in a strong reduction in lateral root numbers in *Arabidopsis* (Shin et al., 2007). Moreover, it has been shown that the bHLH transcription factor BIGPETALp (BPEp) interacts with ARF8 to effect petal growth. This interaction is mediated through the BPEp C-terminal domain and the C-terminal domain of ARF8 (Varaud et al., 2011). The *Arabidopsis* BREVIS RADIX (BRX) transcriptional co-regulator interacts with domain III/IV of ARF5 in yeast two-hybrid assays as well as *in vitro* pull-down assays, and this interaction enhances the transcriptional activation potential of this ARF (Guilfoyle and Hagen, 2012). In another recent report, HaIAA27 was shown to repress the transcriptional activation of the heat shock transcription factor HaHSFA9 in sunflower to repress its activity during seed development. As in the case of the ARFs, auxin also acts to relieve repression of the HaHSFA9 protein (Carranco et al., 2010). Recent data also suggest that post-translational modifications of ARFs may constitute another layer of regulation of auxin signaling outputs (Wang and Estelle, 2014; Hill, 2015). Phosphorylation of ARF7 and ARF19 by BRASSINOSTEROID-INSENSITIVE2 (BIN2) can potentiate auxin signaling output during lateral root organogenesis (Cho et al., 2014). Meanwhile, other previous report shows that BIN2 also phosphorylates ARF2 (Vert et al., 2008). These data suggest that ARF phosphorylation suppresses their interaction with Aux/IAAs, thus enhancing DNA binding and transcriptional activity. In addition, there is a growing body of evidence on the posttranscriptional regulation of ARF transcript abundance by miRNA and **transacting-small interfering RNAs (ta-siRNA)**. While *ARF6* and *ARF8* are targets of miR167 and *ARF10, ARF16*, and *ARF17* are targeted by miR160, *ARF2, ARF3*, and *ARF4* are targets of TAS3 ta-siRNAs in *Arabidopsis* (Rhoades et al., 2002; Williams et al., 2005; Guilfoyle and Hagen, 2007; Lin et al., 2015).

KEY CONCEPT 4 **Transacting-small interfering RNAs (Ta-siRNAs)**

Ta-siRNAs are form of small interfering RNA (siRNA) that represses gene expression through post-transcriptional gene silencing in land plants. They are transcribed from the genome to form a polyadenylated, double-stranded segment of RNA that gets processed further, resulting in a segment of RNA that is 21-nucleotides long. These segments are incorporated into the RNA-induced Silencing Complex and direct the cleavage of target mRNA.

## Roles of *ARFs* in plant growth and developmental processes

The *Arabidopsis* genome encodes 23 ARF proteins (Rademacher et al., 2011) and genetic analyses have shown that individual ARFs control distinct developmental processes based on their loss-of-function mutant phenotypes (Guilfoyle and Hagen, 2007; Rademacher et al., 2012). Although ARFs appear to have unique functions in some contexts, they display overlapping functions in others. For example, both *ARF1* and *ARF2* control leaf senescence and floral organ abscission in *Arabidopsis* (Ellis et al., 2005), while *ARF3* interacts with KANADI proteins to form a functional complex essential for leaf polarity specification (Kelley et al., 2012). A recent study indicated that *ARF3* integrates the functions of *AGAMOUS* (*AG*) and *APETALA2* (*AP2*) in floral meristem determinacy (Liu et al., 2014b), while *ARF4* has been studied primarily for its role in organ polarity (Hunter et al., 2006). However, the *arf3arf4* double mutant plant has reduced abaxial identity in all lateral organs, including leaves (Pekker et al., 2005; Finet et al., 2010). *ARF5* is critically required for embryonic root and flower formation (Hardtke and Berleth, 1998) and embryo patterning and vasculature defects observed in *arf5* mutants are enhanced in *arf5arf7* double mutants (Hardtke and Berleth, 1998). *ARF8* is reported to regulate fertilization and fruit development (Goetz et al., 2006), and *ARF6* and *ARF8* act redundantly in flower maturation (Finet et al., 2010). *ARF19* and *ARF7* act redundantly with in controlling leaf expansion and lateral root growth (Wilmoth et al., 2005). While no phenotypic defects were reported for *arf10* or *arf16* single mutants (Okushima et al., 2005), *arf10arf16* double mutants show a strong auxin phenotype that results in the absence of lateral root formation, which is not observed in neither the *arf10* or *arf16* single mutant (Wang et al., 2005).

In the case of tomato, genetic studies have shown that the mechanism of *ARF* signaling is different to that of *Arabidopsis*. A total of 21 putative functional *SlARFs* have been identified in tomato (Zouine et al., 2014). Although, *SlARF3* RNAi lines do not display phenotypes such as floral organogenes or developmental timing changes (Sessions et al., 1997), *SlARF3* plays multiple roles in tomato development and is involved in the formation of epidermal cells and trichomes (Zhang et al., 2015b). The functional analysis of *SlARF9* indicated that it regulates cell division during early tomato fruit development (DeJong et al., 2015). A recent study confirmed that down-regulation of *ARF6* and *ARF8* by mi167 leads to floral development defects and female sterility in tomatoes. These results indicate that *ARF6* and *ARF8* have conserved roles in controlling growth and development of vegetative and flower organs in dicots (Liu et al., 2014a). *SlARF7* acts as a negative regulator of fruit set until pollination and fertilization have taken place and moderates the auxin response during fruit growth in tomatoes (de Jong et al., 2009). Meanwhile, *SlARF7* mediates cross-talk between auxin and gibberellin signaling during tomato fruit set and development (de Jong et al., 2011). Interestingly, *SlARF4* is involved in the control of sugar metabolism during tomato fruit development (Sagar et al., 2013). In soybeans, the miR167-directed regulation of *GmARF8a* and *GmARF8b* is required for nodulation and lateral root development (Wang et al., 2015). In rice, *OsARF16* and *OsARF12* are required for iron deficiency response by regulating auxin redistribution (Wang et al., 2014; Shen et al., 2015b). *OsARF3* mediates the auxin response during de novo shoot regeneration (Cheng et al., 2013). OsARF19 controls rice leaf angles through positively regulating *OsGH3-5* and *brassinosteroid insensitive 1* (*OsBRI1*) in rice (Zhang et al., 2015a).

## Conclusion and perspectives

During the last 10 years, our understanding of *ARF* regulatory mechanism and their role during model plant growth and development has been greatly improved by forward and reverse genetic approaches. Nonetheless, there are still many gaps in our knowledge and we lack a deep understanding of these regulatory processes. For example, it is still not clear how repressors of *ARFs* regulate gene repression and how other transcription factors and signaling proteins interact with ARF proteins. However, a larger number of candidate genes that are regulated by *ARFs* have been identified both experimentally and through bioinformatics analysis in recent years. Therefore, it will be interesting to understand the function of these candidate genes and regulatory mechanism of some important ARF proteins. In addition, our knowledge of *ARFs* in plant species beyond model plants (typically *Arabidopsis*) is very limited. The great challenge will be to integrate knowledge about ARF regulation of different developmental processes across in plants, and to understand how these processes work in different plant species.

## Author contributions

JZ, SL, and ZX wrote the paper. CH provided some suggestions for the paper.

### Conflict of interest statement

The authors declare that the research was conducted in the absence of any commercial or financial relationships that could be construed as a potential conflict of interest.
